# Development of a capacity‐building curriculum for training informal caregivers of community‐dwelling older persons in a low‐resource setting

**DOI:** 10.1002/alz70858_103487

**Published:** 2025-12-24

**Authors:** Eniola O Cadmus, Oluwaseun O Ajayi, Joy Ojumoola, Aderonke O Alabi, Oluwakayode O Ilugbo, Eme Owoaje, Pamela Teaster, Lawrence A Adebusoye

**Affiliations:** ^1^ University of Ibadan and University College Hospital, Ibadan, Oyo, Nigeria; ^2^ Chief Tony Anenih Geriatric Centre, University College Hospital, Ibadan, Oyo, Nigeria; ^3^ University College Hospital Ibadan, Ibadan, Oyo, Nigeria; ^4^ University of Ibadan, Ibadan, Oyo, Nigeria; ^5^ Center for Gerontology, Virginia Tech, Virginia, VA, USA; ^6^ Department of Community Medicine, College of MedicineUniversity of Ibadan, Ibadan, Oyo, Nigeria

## Abstract

**Background:**

Informal caregivers, usually family, friends, and neighbours, provide essential health and social support in many low and middle‐income countries, including Nigeria. However, most lack formal training to address the diverse needs of older adults, including managing chronic conditions like dementia. This study describes the development of a capacity‐building training curriculum to enhance the skills of informal caregivers of community‐dwelling older persons in southwestern Nigeria.

**Method:**

A multiphase study design was carried out using mixed methods. The initial phase involved a baseline survey among caregivers of older persons. Data were collected over one month by eight trained research assistants using an interviewer‐administered, semi‐structured questionnaire. Thereafter, information obtained was used to develop a culture‐appropriate, context‐specific training curriculum for the training of informal caregivers to provide logistic, social, and emotional support for older persons. Pilot testing of the developed training package was carried out over three days using a volunteer subset of current informal caregivers of community‐dwelling older persons. A pre‐training survey was carried out among the participants to assess their baseline knowledge and practices. Immediately after the training, the same instrument was administered to find out if there were any changes, identify potential challenges and refine the curriculum. Also, the feedback from participants was used to make necessary adjustments and improvements.

**Result:**

The developed curriculum covered a range of topics to ensure caregivers have the necessary skills, knowledge, and compassion to provide quality care. Perceived training needs of the participants included common routine procedures like blood pressure and sugar monitoring, first aid and hazard prevention, knowledge about emergency care, nutritional guidelines, and demystifying cultural myths and misconceptions about ageing. Other training needs include assisting the care recipient with advanced directives and end‐of‐life decision‐making.

**Conclusion:**

This training curriculum will be beneficial and enable improved care coordination skills and competencies of informal caregivers of community‐dwelling older persons in the community and similar low‐resource settings.

